# Unmet needs and quality of life burden in chronic myeloid leukemia: results from a nationwide Italian real-world survey

**DOI:** 10.3389/fonc.2026.1846801

**Published:** 2026-07-16

**Authors:** Elisabetta Abruzzese, Mario Annunziata, Felice Bombaci, Antonella Russo Rossi, Anna Galante, Alessandra Misto, Diletta Valsecchi, Evgeniia Gushchina, Carmen Fava

**Affiliations:** 1 Department of Hematology, S Eugenio Hospital, Tor Vergata University, ASL Roma2, Rome, Italy; 2 Division of Hematology, Azienda Ospedaliera Rilievo Nazionale (AORN) Cardarelli, Naples, Italy; 3 AIL- Associazione Italiana Contro Leucemie, Linfomi, Mieloma, Rome, Italy; 4 Hematology and Stem Cell Transplantation Unit, AOU Consorziale Policlinico, Bari, Italy; 5 Bianco Airone A.P.S., Rome, Italy; 6 Novartis Farma S.p.A, Milano, Italy; 7 Department of Clinical and Biological Sciences, University of Turin, Turin, Italy

**Keywords:** chronic-phase, health surveys, leukemia, myeloid, quality of life, tyrosine kinase inhibitors

## Abstract

**Background:**

Optimizing tyrosine kinase inhibitor (TKI) therapy in chronic myeloid leukemia (CML) requires careful consideration of patient quality of life (QoL), which significantly influences treatment adherence and long-term outcomes. Long-term treatment can be associated with persistent symptoms that may negatively impact QoL, adherence, and patient satisfaction. This nationwide Italian survey explored patient experiences, treatment patterns, and factors influencing clinical decision-making in CML, with the aim of better understanding unmet needs and potential barriers to optimal care.

**Methods:**

We conducted a cross-sectional survey involving 146 adult patients with CML. A structured questionnaire, developed collaboratively by hematologists and patient association representatives, assessed patient-reported outcomes (PROs), treatment-related burden, and care satisfaction. Analyses were descriptive and exploratory.

**Results:**

Despite the availability of effective therapies, a substantial proportion of patients reported impaired QoL due to both disease and treatment-related factors. While most patients reported overall satisfactory QoL, 26% described low or very low QoL. The burden was particularly evident in specific patient subgroups and in later lines of therapy, reflecting a significant physical and psychological impact of CML and its management. Fatigue, weakness, and muscle pain were the most frequently reported symptoms, often persistent and affecting daily functioning. Female patients reported a higher symptom burden and greater psychological distress compared to males. Despite high overall satisfaction with healthcare professionals, relevant unmet needs were identified, particularly regarding information on supportive services, long-term disease management, and fertility-related issues. Treatment-related adverse events were common and frequently chronic, suggesting a substantial cumulative burden over time.

**Conclusions:**

Despite the effectiveness of TKIs, patients with CML continue to experience a meaningful and multidimensional burden, particularly in later treatment lines and specific subgroups. These findings highlight the importance of greater attention to patient-centered management in CML, with treatment decisions carefully balancing the benefits and limitations of available therapeutic options to optimize both clinical outcomes and quality of life.

## Introduction

Tyrosine kinase inhibitors (TKIs) have played a pivotal role in the substantial progress made in the treatment of hematological malignancies and solid tumors. The advent of these targeted therapies has resulted in a paradigm shift in the long-term prognosis and life expectancy of patients with chronic myeloid leukemia (CML) diagnosed in the chronic phase (1–3).

The use of first- and second-generation TKIs (imatinib, bosutinib, dasatinib, and nilotinib) has dramatically reduced CML-related mortality. However, patients may develop resistance and/or intolerance to TKIs, leading to reduced adherence, worse clinical outcomes and reduced overall survival (4).

Thanks to new TKIs, such as ponatinib and asciminib, treatment options have been expanded for these patients. Ponatinib is a pan–BCR::ABL1 TKI with potent activity against native and mutated BCR::ABL1 kinases, including T315I (5); asciminib is a first-in-class BCR::ABL11 inhibitor specifically targeting the ABL myristoyl pocket (STAMP) (6, 7).

Both patients and healthcare professionals aim to prevent disease progression and aspire to a treatment-free remission (TFR) but, unfortunately, only 25% of patients with CML can achieve durable TFR with careful monitoring (3, 8–10).

A three-pronged strategy integrating efficacy, safety, and cost should inform TKIs selection, contingent upon patient characteristics, healthcare resources, and therapeutic objectives (11, 12).

According to the European Leukemia Net (ELN) guidelines on CML, the main goal of TKIs therapy is Major Molecular Response (MMR); Deep Molecular Response (DMR) and TFR when feasible (13). Clinicians tend to prioritize therapy effectiveness and achieve response targets, such as DMR and TFR, while patients are more concerned about potential side effects in all treatment lines (14). Low-grade chronic toxicities resulting from TKI treatment, especially if persistent, can adversely affect quality of life (QoL), making patients less inclined to strictly adhere to the prescribed long-term treatment plan (15–18).

Treatment adherence and persistence are a constant challenge for patients requiring long-term treatment. Patients who strictly adhere to the prescribed treatment plans are more likely to achieve DMR. Specifically, patients with a >90% adherence rate to imatinib were significantly more likely to experience a DMR within six years (19). In contrast, non-compliance with imatinib therapy has been identified as the most significant contributing factor to inadequate response and molecular relapse (20). Patients with CML in the chronic phase (CML-CP) who adhere to their prescribed medication regimen and achieve MMR, can expect survival rates comparable to those observed in the general age-matched population (11, 21, 22).

It is therefore important to not only communicate the potential for curing CML and resuming a normal life, but also to thoroughly explore, step by step, the long-term implications of treatments (23).

Patients benefit from receiving clear information about their disease, which helps them feel prepared to engage in treatment decisions. Clinicians can support this process by ensuring patients understand their needs and have the opportunity to express their concerns about outcomes and expectations, fostering effective two-way communication and shared decision-making (24, 25). The authors agreed that engaging patients in meaningful ways and providing them with the necessary tools to track and report their symptoms and perceptions of well-being is an important part of the communication to build a therapeutic alliance.

A recent study based on an online survey of patients and physicians in 11 countries highlighted the critical importance of effective communication and shared decision-making in CML-CP management (14). The study revealed discrepancies between patients and physicians, especially regarding the balance between the efficacy and tolerability of therapy. Notably, only 25% of patients reported discussing and making treatment decisions with their physician (14).

Our study aimed to gain deeper insight into the experiences of individuals with CML in Italy, enhancing our understanding of the disease from the affected individuals’ perspective. The present study had three goals: first, to characterize Italian patients with CML-CP; second, to evaluate their experiences with the disease, and third delineate their unmet clinical and care needs.

## Materials and methods

### Study design

A nationwide, cross-sectional pharmaceutical market research survey conducted in Italy, in accordance with EphMRA, ESOMAR, and GDPR standards, evaluated the treatment-related issues, the disease burden, the perceived overall QoL and care satisfaction among patients with CML-CP.

### Questionnaire development

A multidisciplinary Working Group, including CML hematologists, a QoL specialist, and patient advocacy representatives, guided survey development. An initial item pool was generated via literature review and refined for content validity. Patient associations (Associazione Italiana contro Leucemie, Bianco Airone A.P.S.) assessed clarity and patient-centeredness. Feedback was collected from the participating physicians, and the final survey comprised 32 multiple-choice questions (**Supplementary Table 1**, **ST1**) addressing: 1) experiences and challenges of living with CML; 2) unmet needs in management; and 3) treatment practices, focusing on targeted therapies. Variables included sociodemographic characteristics of the participants, comorbidities, clinical history, treatment patterns. Patient-Reported Outcome Measures (PROMs) and Patient-Reported Experience Measures (PREMs) assessed disease impact, symptoms, satisfaction with medical and social support, treatment, and care.

The survey, available through secure CAWI online or in paper format, required about 10–15 minutes to complete. Where indicated, Likert-type scales allowed respondents to indicate the extent of their agreement or disagreement with statements.

### Survey administration and recruitment

Adults with CML (≥18 years) were recruited from November 2024 to April 2025 either through patient advocacy outreach or by clinicians following routine clinical visits. All participants were invited to complete, at their discretion, the structured questionnaire available in printed format or as an online version. Eligible participants had confirmed CP-CML and completed the survey. Exclusions included other hematologic malignancies or pharmaceutical employment. Informed consent was obtained, and participants were acknowledged for their contribution with a fee in line with fair-market standards.

### Statistical analysis

Quantitative data were screened for outliers, and descriptive analyses summarized variable distributions. Satisfaction with medical support and information was evaluated via mean item scores. Group comparisons by therapy line, age (≤60 vs >60), and sex were reported descriptively.

The overall prevalence was calculated across the entire study population, while subgroup percentages were derived independently within each sex-specific or therapy-line subgroup, providing a more detailed view of the distribution across different patient categories. No formal statistical tests were conducted; results are exploratory and hypothesis-generating.

## Results

### Population characteristics

A total of 146 patients (7 newly diagnosed) with CML were enrolled, representing a satisfactory sample size considering the disease epidemiology. Of these, 104 participants were referred by physicians and 42 were recruited through patient advocacy organizations. The cohort included 92 males (63%) and 54 females (37%), with participants distributed across the northern (29%), central (47%), and southern (25%) regions of Italy. Fifty-six patients were younger than 60 years old and 69 were > 60; 21 participants did not provide age details. Most patients had upper secondary or higher education. Regarding employment, 55% were employed, 42% retired, and 3% never worked. Most were married/partnered (86%).

Most respondents reported living with a partner or other family members, whereas 12% declared living alone (**Table 1**).

**Table 1:** Sociodemographic characteristics of the participants. .

Characteristics	Total (N = 146)
Gender, n (%)	
Male	92 (63%)
Female	54 (37%)
Area, n (%)	
Northern Italy	42 (28,77%)
Central Italy	68 (46,57%)
Southern Italy	36(24,66%)
Age in years, n (%)	
≤50	25 (17%)
51-60	31 (21%)
61-65	16 (11%)
66-70	25 (17%)
>70	28 (19%)
Unknown	21 (14%)
Work status, n (%)	
Employed	81 (55%)
Retired	61(42%)
Never worked	4 (3%)
Marital status, n (%)	
Partner or Married	125 (86%)
Single	17 (12%)
Unknown	4 (2%)
Education, n (%)	
Lower secondary education	38 (26%)
Upper secondary education	64 (44%)
Bachelor's degree or master's degree	44 (30%)
Comorbidities, n (%)	
No	52 (36%)
Yes	94 (64%)
CML Phase, n (%)	
Chronic phase	130 (89%)
Accelerated phase	3 (2%)
Blast phase	3 (2%)
Unreported	10 (7%)
Therapy Line, n (%)	
New diagnosis	7 (5%)
First line (Imatinib)	75 (51%)
Second line (Bosutinib, Nilotinib, Dasatinib)	39 (27%)
Third line (Ponatinib Asciminib)	5 (4%)
Fourth line and subsequent	8 (5%)
Unknown	12 (8%)
Side effect, n (%)	
Yes	58 (40%)
No	86 (59%
Unreported	2 (1%)
TKIs tolerance /resistance, n (%)	
Intolerance	58 (40%)
Resistance	22 (15%)
Nothing	66 (45%)

Comorbidities were common, reported by 64% of patients and increasing with age (46% ≤60 years vs. 77% >60). Frequent conditions included hypertension (55%), dyslipidemia (47%), diabetes (28%), and cardiovascular disease (21%). Nearly half (49%) underwent additional therapeutic interventions alongside CML treatment. Comorbidities are summarized in **Table 2** (multiple choice). Participants had lived with CML for a mean of 15 years (diagnosed between 1990 and 2024; average year of diagnosis 2009; standard deviation 11.7 years), with most (89%) in the chronic phase. Over half reported asymptomatic, incidentally diagnosed disease, while 10% received their diagnosis following an emergency department admission.

**Table 2:** Reported comorbidities.

Comorbidities	n (94)	% (Rounded)
Diabetes	26	28%
Dyslipidemia	44	47%
Obesity	9	10%
Hypertension	52	55%
Cardiovascular Disease	20	21%
Respiratory Disease	13	15%
Chronic Kidney disease	7	7%
Hepatic failure	2	2%
Autoimmune Disease	9	10%
Anxiety, depression	12	13%
Neurologic disease	3	3%
Ocular disease	10	11%
Others	12	13%

Hematologists were the primary specialists consulted, with 58% of patients reporting follow-up with more than one hematologist. Other healthcare professionals involved in the CML care pathway included primary care physicians (54%), cardiologists (50%), nurse practitioners (27%), and psychologists (18%).

### Overall QoL and perceived disease impact

While 74% of the study participants reported an overall satisfactory QoL, 26% still considered it to be low or very low. Gender-stratified analysis showed that a greater proportion of women reported low QoL compared to men (27% vs. 16%).

Among patients receiving third-line therapy, 46% reported a low or very low quality of life. The corresponding proportions in the first- and second-line therapy groups were 21% and 28%, respectively. Even if expressed as percentages, these findings are not directly comparable due to unequal sample sizes across treatment lines.

Most respondents reported muscle pain (59%), weakness or tiredness (73%), and chronic fatigue (65%). Chronic fatigue was particularly prevalent among patients receiving second- and third-line therapies, affecting 92% of this subgroup. Gender-stratified analysis showed that women experienced higher prevalence of muscle pain (55% vs. 49%), weakness (76% vs. 63%), and chronic fatigue (76% vs. 51%), compared to men.

The disease is also associated with a psychological burden, particularly among patients receiving second- and third-line therapies. A substantial proportion of the respondents reported an overall decline in multiple life domains, including their relationships with themselves (29%), families (27%), and social relationships (26%). Deterioration was also noted in psychological well-being (28%), sexual activity (25%), and the ability to make future plans (22%).

Negative impacts were reported in professional life, leisure activities, physical exercise, sports participation, and financial well-being, although these were less frequent, affecting 15–20% of respondents.

The psychological burden of CML encompasses emotional distress related to illness, the ongoing need for medication, uncertainty regarding prognosis, fear of disease progression and the indirect impact of the disease on the family. Women reported higher levels of anxiety concerning health deterioration and uncertain survival than men (42% vs. 21% and 30% vs. 21%, respectively). Additionally, the fear of losing autonomy and becoming dependent on others was more pronounced in women (9% vs. 5%) and in patients receiving third-line therapy (15% vs. 3% in first- and second-line therapies).

The majority of patients reported traveling 30–60 minutes to reach their treatment center for follow-up visits and examinations, typically four to five times per year. Patients on later lines of therapy required more intensive monitoring, with up to 10 visits annually. Regarding access to medication, 78% of patients obtained their CML medication from hospital pharmacies at intervals of 1–3 months, 18% from community pharmacies, and only 2% reported access through home delivery services.

### Treatment-related issues

Among the 146 patients surveyed, 58 (40%) reported experiencing adverse effects related to CML-CP therapy, categorized as mild (n = 22), moderate (n = 25), or severe (n = 11). The number of side effects varied: 22% of patients reported at least two, 31% reported three to five, and 27% experienced six or more adverse effects. Side effects were reported by 19 out of 75 patients who declared receiving first-line therapy, 20 out of 39 who indicated receiving second-line therapy, and 9 out of 13 who declared receiving third- or subsequent-line therapy.

Notably, severe intolerance, whether side effects or adverse reactions, was more common during third- and subsequent-line therapy (67% vs. ≤15% in earlier lines).

Among the 58 patients experiencing side effects, 21% reported a duration of a few days, 16% a few weeks, 21% a few months, 7% reported persistence for years, and 36% described side effects as a constant feature of therapy.

**Table 3** summarizes the TKIs–related adverse effects most frequently reported by surveyed patients (multiple-choice question).

**Table 3:** Adverse events reported by the 58 patients who self-reported them.

Adverse Events	%
Nausea	24%
Abdominal pain	9%
Diarrhea	28%
Muscle spasms	40%
Headache	14%
Fatigue	43%
Weight gain	36%
Ocular bleeding/conjunctival hyperemia	19%
Periorbital edema	33%
Rash	26%
Skin discoloration	9%
Skin thinning	21%
Cardiovascular adverse effect	21%
Respiratory adverse effect	9%
Others	22%

Treatment adherence was high, with 96% of respondents reporting full compliance with the prescribed regimen. However, when asked further questions, 25% of patients admitted occasionally missing doses, and 8% reported non-adherence when asymptomatic.

Therapy resistance increased with treatment progression. Among patients on second-line therapy, 38% developed resistance, with half of these cases occurring within nine months of treatment initiation. In contrast, 62% of patients on third- or later-line therapies exhibited resistance, which most commonly developed within one year of starting the most recent treatment line.

### Overall care satisfaction

Healthcare professionals were rated highly for the provision of information and support, with a mean score of 6.0 on a seven-point Likert scale. Patients reported the highest perceived support at the time of diagnosis (mean 6.2), higher than during therapy transition (mean 5.5). Satisfaction with involvement in treatment decision-making was slightly lower among patients receiving third- or subsequent-line therapies (mean 5.7) than among those receiving earlier lines (mean 5.9). The lowest scores were obtained for information on available services and care resources, as well as procreation topics for younger people.

The support needed varies depending on the therapy and age of the patient.

Approximately 40% of first- and second-line patients reported that they did not need support, while 15% of third- and subsequent-line patients required support for daily activities, despite their age.

The need for support by family members in disease management was higher (approximately 25%), with the highest percentage (36%) among respondents aged > 60 years.

Finally, more than half of the respondents reported receiving emotional support from their partners, family members, and friends when needed.

## Discussion

CML is often considered a model of successful targeted therapy, with near-normal life expectancy for many patients in the chronic phase. However, our nationwide real-world survey highlights that, despite the availability of effective TKI therapy, the disease and its treatment exert a substantial and multifaceted impact on patients’ QoL, in keeping with the experience of other chronic conditions.

Although validated QoL assessment tools provide valuable information about patient health, their integration with patient-reported data significantly improves the clinical relevance and effectiveness of healthcare (25). To this end, the authors, together with CML patients, developed the survey presented in this publication, designed to capture the full spectrum of patient-reported outcomes, like self-reported health status, symptoms, and functioning (PROs) and patient-reported experience including communication, support, and system responsiveness (PREs), as conventional toxicity scales may underestimate the real-life burden of low grade chronic adverse events (14).

TKIs, though proven to be therapeutically effective, can induce acute and/or chronic side effects over time, with all agents causing hematological toxicity and mostly off-target adverse events observed in real-world studies (26). Importantly, conventional toxicity scales may underestimate the impact of low-grade adverse events on treatment adherence and overall well-being (26). The present study showed that about 40% of patients experienced treatment side effects, with approximately 25% having moderate to severe side effects and 23% reporting three or more effects. Notably, a higher symptom burden was reported among women, suggesting potential gender-specific differences in disease perception and impact.

Clinicians have increasingly recognized the consequences of low-grade, yet protracted toxicity associated with TKIs. An Italian analysis described a (yearly) growth in the number of patients treated with TKIs as third-line or later therapy, with a concomitant increase in healthcare costs, due to hospitalization (27). In our cohort, side effects persisted in 14% of patients, indicating that chronic toxicity may represent a relevant long-term burden, particularly in patients receiving multiple lines of therapy. These data highlight the need for continued attention to patients undergoing prolonged treatment exposure.

From a clinical perspective, our findings suggest that the assessment of all toxicities, including those considered mild, should be systematically integrated into routine practice, given their potential impact on QoL and long-term treatment adherence. Although adherence to therapy in our study was higher than expected, likely reflecting effective patient–physician communication and established long-term disease management strategies, it remains important for healthcare professionals to closely monitor patient-reported outcomes (PROs), particularly among younger patients and those more affected by treatment-related symptoms, to prevent deviation from the prescribed regimen. The findings about adherence highlight the necessity of expanding the assessment beyond a singular, direct inquiry regarding treatment intake. Clinicians are advised to incorporate additional, targeted confirmation questions—such as inquiries about the frequency of missed doses, contextual factors affecting adherence, and patient behaviors during asymptomatic periods—to achieve a more precise and nuanced comprehension of real-world medication use.

It is also important to consider patient-specific factors. In our study, a significant proportion of patients, including nearly 80% of those over 60 years of age, reported at least one comorbidity, which may further influence treatment tolerability and clinical decision-making. Consequently, variations in TKI safety profiles should be carefully weighed when selecting treatment options for patients with CML-CP, particularly in the presence of cardiovascular or metabolic comorbidities (11). On the other hand, subsequent deterioration in treatment adherence has also been shown to be related to low satisfaction with care and support (28). In our cohort, patients reported high levels of satisfaction with healthcare professionals, indicating that hematologists provide strong support to CML patients, particularly in the early stages of the disease, suggesting that communication and clinical support are generally well established. However, the persistence of unmet needs despite high satisfaction highlights the complexity of patient experience and indicates that additional supportive strategies may be required.

In patients with hematological malignancies, an unpredictable disease course is common, with worsening and resistance to therapy occurring during an otherwise stable course of the disease (29). Although clinicians intend to provide reassurance at the time of diagnosis, as recognized by 80% of those interviewed, patients may subsequently experience uncertainty or feel isolated, especially if the treatment line changes. This could result in patients feeling uncertain about sharing their concerns with their healthcare team. This underscores the importance of transparent and continuous communication, including discussions about potential treatment failure and disease evolution. Effective communication remains essential at all stages of the disease to foster strong physician-patient relationships and improve satisfaction, clinical, and psychosocial outcomes. Our survey indicates that women often need additional reassurance about potential health deterioration, uncertain life expectancy, and long-term prognosis, while younger patients are more concerned about fertility and pregnancy. This highlights the need for tailored communication and dedicated counseling, particularly for patients of reproductive age, as well as for hematologists to develop strong knowledge and communication skills when addressing fertility-, pregnancy-, and sexual health–related concerns (30, 31). Patients should be informed about the risks associated with unplanned pregnancies including potential fetal complications, as well as the risks associated with CML treatment interruption. They should also be advised that a controlled pregnancy may be possible under appropriate clinical conditions (13). Although uncertainties remain, increasing awareness among physicians and patients, together with recent recommendations, has improved the planning of both spontaneous and medically assisted pregnancies, as well as the management of planned or unplanned conception (31–33).

Finally, our results show that patients receive assistance from their partners or family members, with a limited proportion requiring continuous assistance for daily activities. While caregiver burden appears lower compared to other chronic conditions (only one-third of patients require ongoing assistance for disease management and daily living activities), their involvement remains an important component of comprehensive care. Increased attention to caregiver engagement and educational initiatives for healthcare professionals may further enhance patient support.

The relevance of our work lies primarily in the assessment of QoL outside the setting of a pharmacological clinical trial, while also capturing the patient perspective within an unselected, real-world CP-CML population.

Another key strength of our research is the early and active involvement of patient associations, in study design and dissemination. The co-creation of a shared questionnaire with patient representatives contributed to capturing a broad and relevant spectrum of patient experiences, reinforcing the patient-centered nature of the study, and ensuring relevance, accuracy, and meaningful impact. This participatory approach supports the relevance and applicability of the findings in real-world clinical practice, empowering individuals as co-creators of value-based care rather than passive subjects.

This study is subject to the usual methodological limitations inherent in survey-based research. These include potential selection bias, recall bias, and limited generalizability to the broader population affected by CML-CP. In addition, the cross-sectional design limited the ability to draw causal inferences between the observed variables. These limitations must be considered when interpreting the results and their implications for clinical practice and future research.

Despite these limitations, our findings underscore the clinical importance of maintaining careful attention to patient-centered management, with treatment decisions balancing efficacy, tolerability, and patient preferences. In this context, the systematic integration of PROs may facilitate more informed and individualized therapeutic strategies, particularly in patients receiving long-term or multiple lines of therapy. Overall, this study provides meaningful insights into the real-world experience of patients with CML and offers a foundation for improving patient care and informing future research.

## Data Availability

The raw data supporting the conclusions of this article will be made available by the authors, without undue reservation.
