# Supplementary material for: Unmet needs and quality of life burden in chronic myeloid leukemia: results from a nationwide Italian real-world survey
Source: Front Oncol. 2026 Jul 16;16:1846801. doi: 10.3389/fonc.2026.1846801 (PMC13422165; doi:10.3389/fonc.2026.1846801)
Supplement: Supplementary file 1 [file Table1.docx]

# Supplementary Material

# Table 1. Full Questionnaire (Italian)

## Q1 – Lei ha ricevuto una diagnosi di leucemia mieloide cronica?

| Opzioni di risposta | □ Sì  □ No |
| --- | --- |

## Q1_2 – In quale fase della malattia si trova?

| Opzioni di risposta | □ Fase cronica  □ Fase accelerata  □ Fase blastica  □ Non saprei |
| --- | --- |

## Q2_1 – Quale linea di trattamento sta seguendo attualmente?

| Opzioni di risposta | □ Nuova diagnosi  □ Prima linea  □ Seconda linea  □ Terza linea  □ Quarta linea o successive □ Non saprei |
| --- | --- |

## Q2_2 – Quale terapia sta seguendo ATTUALMENTE?

| Opzioni di risposta | □ Imatinib (generico) □ Glivec (imatinib) □ Tasigna (nilotinib) □ Sprycel (dasatinib) □ Bosulif (bosutinib) □ Iclusig (ponatinib) □ Scemblix (asciminib) □ Non sto più seguendo un trattamento farmacologico  □ Altra terapia farmacologica (specificare)  □ Non saprei |
| --- | --- |

## Q3 – Soffre di altre patologie o problemi di salute?

| Opzioni di risposta | □ Diabete □ Dislipidemia □ Obesità □ Colesterolo/trigliceridi elevati □ Ipertensione □ Malattie cardiovascolari □ Malattie respiratorie (asma, BPCO, pneumopatie) □ Insufficienza renale □ Insufficienza epatica □ Epatite B/C □ Malattie autoimmuni □ Ansia/Depressione □ Malattie neurologiche □ Disturbi visivi □ Altre malattie (specificare) □ No, nessuna concomitante |
| --- | --- |

## Q3a – Assume altre terapie, oltre a quella per la LMC?

| Opzioni di risposta | □ Sì: numero di terapie ___ □ No |
| --- | --- |

## Q4 – Ha effettuato trapianto di cellule staminali?

| Opzioni di risposta | □ Sì  □ No |
| --- | --- |

## Q5 – In che anno ha ricevuto la diagnosi?

| Opzioni di risposta | Spazio per l’anno o età alla diagnosi |
| --- | --- |

## Q6 – Come ha scoperto di avere la LMC?

| Opzioni di risposta | □ Esami del sangue volontari □ Esami consigliati dal MMG per altra condizione sospetta □ Accesso in PS per sintomi acuti □ Esami su indicazione del MMG per sintomi non acuti □ Altro (specificare) |
| --- | --- |

## Q7 – Quali parole descrivono ciò che ha provato alla diagnosi?

| Opzioni di risposta | □ Accettazione □ Smarrimento □ Paura □ Ansia/angoscia □ Indifferenza □ Disprezzo □ Rabbia □ Sorpresa □ Forza □ Stanchezza □ Negazione □ Sfiducia □ Rassegnazione □ Fiducia □ Altro (specificare) |
| --- | --- |

## Q8 – Come valuta la sua qualità di vita?

| Opzioni di risposta | Scala visiva da 'Qualità di vita pessima' a 'Qualità di vita ottima' |
| --- | --- |

## Q9 – Quali sono gli aspetti negativi?

| Opzioni di risposta | □ Sintomi della malattia □ Effetti collaterali □ Dover assumere terapia □ Essere malato/a □ Incertezza sul tempo a disposizione □ Dipendenza dagli altri □ Non riuscire a stare con gli altri □ Non poter pianificare □ Sentirsi soli/non capiti □ Paura del peggioramento □ Rinuncia alle attività □ Incertezza della cura □ Nessun aspetto negativo |
| --- | --- |

## Q10/Q16 – Impatto sulle sfere di vita (1–13)

| Opzioni di risposta | Scala 1–7 per 13 aree (famiglia, lavoro, economia, psicologia, ecc.) |
| --- | --- |

## Q11 – Condizione fisica

| Opzioni di risposta | Scala 0–10 per: dolori muscolari, affaticamento, stanchezza cronica |
| --- | --- |

## Q12 – Parole che descrivono come si sente oggi

| Opzioni di risposta | Stessa lista opzioni Q7 |
| --- | --- |

## Q12a – Intolleranza alla terapia

| Opzioni di risposta | □ Sì  □ No |
| --- | --- |

## Q12_1 – Giorni di assenza per effetti collaterali

| Opzioni di risposta | □ Numero giorni ___ □ Non lavoro/sono pensionato |
| --- | --- |

## Q12b – Gravità intolleranza

| Opzioni di risposta | □ Lieve  □ Moderata  □ Severa |
| --- | --- |

## Q12c – Effetti collaterali sviluppati

| Opzioni di risposta | □ Nausea □ Crampi □ Diarrea □ Mal di testa □ Stanchezza cronica □ Gonfiore occhi □ Aumento peso □ Emorragie oculari □ Occhi gonfi □ Eruzioni cutanee □ Scolorimento pelle □ Assottigliamento pelle □ Problemi cardiaci □ Problemi respiratori □ Mal di pancia □ Altro (specificare) |
| --- | --- |

## Q12d – Durata effetti collaterali

| Opzioni di risposta | □ Qualche giorno □ Qualche settimana □ Qualche mese □ Qualche anno □ Non terminati |
| --- | --- |

## Q13 – Resistenza alla terapia

| Opzioni di risposta | □ Sì  □ No |
| --- | --- |

## Q13a – Tempo alla resistenza

| Opzioni di risposta | □ 3 mesi □ 6 mesi □ 9 mesi □ 1 anno □ 2 anni □ >2 anni □ Non ricordo |
| --- | --- |

## Q14 – Compresse e frequenza

| Opzioni di risposta | Spazio per numero compresse e volte/die |
| --- | --- |

## Q15 – Dimentica la terapia?

| Opzioni di risposta | □ Sì  □ No |
| --- | --- |

## Q16 – Non assume terapia quando sta bene?

| Opzioni di risposta | □ Sì □ No |
| --- | --- |

## Q17 – Difficoltà aderenza

| Opzioni di risposta | □ Sì, motivare □ No |
| --- | --- |

## Q18 – Frequenza difficoltà

| Opzioni di risposta | □ Mai □ Raramente □ Qualche volta □ Spesso □ Sempre |
| --- | --- |

## Q19 – Dove ritira la terapia

| Opzioni di risposta | □ Farmacia ospedaliera □ Territoriale/ASL □ Consegna domicilio □ Altro |
| --- | --- |

## Q20 – Frequenza ritiro

| Opzioni di risposta | □ Ogni mese □ Ogni 2 mesi □ Ogni 3 mesi □ Ogni 4–5 mesi □ Ogni 6 mesi □ Meno frequentemente |
| --- | --- |

## Q21 – Supporto dei medici

| Opzioni di risposta | Scala 1–7 per 6 fasi |
| --- | --- |

## Q22 – Medici che la seguono

| Opzioni di risposta | Elenco ematologo, MMG, cardiologo, ecc. con numero |
| --- | --- |

## Q23 – Tempo per raggiungere il centro

| Opzioni di risposta | □ <30 min □ ~1 h □ ~1.5 h □ ~2 h □ >2 h |
| --- | --- |

## Q24 – Visite annue

| Opzioni di risposta | □ <3 □ 4–5 □ 6–7 □ 8–9 □ 10 □ >10 |
| --- | --- |

## Q25 – Supporto delle persone

| Opzioni di risposta | Per partner/familiari/amici/assistenti:  □ Al bisogno  □ Continuativo  □ Nessuno |
| --- | --- |

## Q26 – Tipo di supporto

| Opzioni di risposta | □ Aiuto quotidiano □ Aiuto gestione malattia □ Supporto emotivo □ Nessun bisogno |
| --- | --- |

## Q26_1 – Giorni assenza caregiver

| Opzioni di risposta | □ Numero giorni ___ □ Non ho caregiver |
| --- | --- |

## Q27 – Soddisfazione

| Opzioni di risposta | Scala visiva per 7 aree |
| --- | --- |

## Q27a – Coinvolgimento e supporto psicologico

| Opzioni di risposta | Scala visiva |
| --- | --- |

## Q28 – Bisogni aggiuntivi

| Opzioni di risposta | □ Più informazioni □ Più coinvolgimento □ Supporto psicologico □ Confronto pazienti □ Permessi lavoro □ Esenzione visite □ Maggiore sensibilità □ Comunicazione clinica □ Supporto economico □ Altro |
| --- | --- |

## Z1 – Anno di nascita

| Opzioni di risposta | Campo libero |
| --- | --- |

## Z2 – Regione

| Opzioni di risposta | Elenco 20 regioni italiane |
| --- | --- |

## Z3 – Titolo di studio

| Opzioni di risposta | □ Nessuno □ Elementare □ Media □ Biennio superiore □ Quinquennio superiore □ Laurea □ Post-laurea |
| --- | --- |

## Z4 – Condizione lavorativa

| Opzioni di risposta | □ Lavoro □ Non ho mai lavorato □ Pensione |
| --- | --- |

## Z5 – Stato civile

| Opzioni di risposta |  |
| --- | --- |

## Z6 – Figli

| Opzioni di risposta | □ Sì  □ No |
| --- | --- |

## Z7 – Composizione nucleo familiare

| Opzioni di risposta | □ Solo/a □ Con partner □ Con figli □ Altro |
| --- | --- |
